# Delayed functional therapy after acute lateral ankle sprain increases subjective ankle instability – the later, the worse: a retrospective analysis

**DOI:** 10.1186/s13102-021-00308-x

**Published:** 2021-08-06

**Authors:** Christian Raeder, Janina Tennler, Arthur Praetorius, Tobias Ohmann, Christian Schoepp

**Affiliations:** 1 Clinic for Arthroscopic Surgery, Sports Traumatology & Sports Medicine, BG Klinikum Duisburg, Duisburg, Germany; 2 Research Department, BG Klinikum Duisburg, Duisburg, Germany

**Keywords:** Epidemiology, Functional rehabilitation, Ankle injury, Ankle instability, FADI

## Abstract

**Background:**

The lateral ankle sprain (LAS) is one of the most common injuries in everyday and sports activities. Approximately 20–40 % of patients with LAS develop a chronic ankle instability (CAI). The underlying mechanisms for CAI have not yet been clearly clarified. An inadequate rehabilitation after LAS can be speculated, since the LAS is often handled as a minor injury demanding less treatment. Therefore, the aims of this retrospective study were to determine the CAI rate depending on age and sex and to identify possible determinants for developing CAI.

**Methods:**

Between 2015 and 2018 we applied the diagnostic code “sprain of ankle” (ICD S93.4) to identify relevant cases from the database of the BG Klinikum Duisburg, Germany. Patients received a questionnaire containing the Tegner-Score, the Cumberland Ankle Instability Tool (CAIT) and the Foot and Ankle Disability Index. Additionally, there were questions about the modality and beginning of therapy following LAS and the number of recurrent sprains. There was a total of 647 completed datasets. These were divided into a CAI and non-CAI group according to a CAIT cut-off-score with CAI ≤ 24 and non-CAI > 24 points, representing one out of three criteria for having CAI based on international consensus.

**Results:**

The overall CAI rate was 17.3 %. We identified a higher CAI rate in females and within the age segment of 41 to 55 years. A later start of therapy (> 4 weeks) after acute LAS significantly increases ankle instability in CAIT (*p* < .05). There was a significantly higher CAIT score in patients having no recurrent sprain compared to patients having 1–3 recurrent sprains or 4–5 recurrent sprains (*p* < .001).

**Conclusions:**

Females over 41 years show a higher CAI rate which implies to perform specific prevention programs improving ankle function following acute LAS. A delayed start of therapy seems to be an important determinant associated with the development of CAI. Another contributing factor may be a frequent number of recurrent sprains that are also linked to greater levels of subjective ankle instability. Therefore, we would recommend an early start of functional therapy after acute LAS in the future to minimize the development of CAI.

## Background

The lateral ankle sprain (LAS) is one of the most common musculoskeletal injuries in everyday and sports-related activities [1]. In the general public, an incidence rate between 2.2 and 7 LAS per 1000 person-years has been reported [2]. Up to 70 % of the general population state having suffered from at least one LAS during their lifetime. In addition, there is a twofold increased risk of re-injury in the year following the initial injury [3]. In sports, an incidence rate ranging between 0.88 and 7 LAS per 1000 exposures has been reported, with indoor and court sports showing the greatest injury risk [4]. There is a high rate of recurrent ankle sprains ranging between 12 and 70 % [5, 6] with a five times increased risk of re-injury [7]. This has been shown in particular to be an important contributor for the development of chronic ankle instability (CAI) [1, 5]. Minimizing the recurrence rate should therefore be an important goal of functional therapy after acute LAS.

The medical treatment, work loss as well as a loss of productivity lead to high socioeconomic costs, especially with recurrent sprains and long-term problems [1]. Epidemiological studies have shown that the total costs of a LAS in the European Union range between 800 € and 1100 € [1]. In the Netherlands, productivity loss due to absence from work was responsible for up to 80 % of the total costs of a LAS [8]. In 2019, there were 60,000 LAS in Germany leading to a total of 550,000 lost workdays (on request at the DGUV from 13.08.2020; DGUV, German Statutory Accident Assurance). Approximately 20–40 % of patients with a LAS will develop a CAI which is defined as a continuum of mechanical and/or functional instability resulting in subjective instability, recurrent sprains and persistent pain lasting > 1 year after the initial LAS [6, 9, 10]. Consequently, the LAS is a serious injury of high social relevance that requires adequate treatment to prevent negative long-term effects and chronic symptoms.

One possible explanation for the high instability rate is an insufficient rehabilitation and/or a too early return to intense sports and workloads [3, 11]. Up to now the LAS is still handled as a minor injury that will resolve quickly with limited treatment although the primary LAS is often the start point for severe and long-lasting symptoms [12, 13]. Several risk factors for incurring a LAS have been proposed such as a younger age, a history of recurrent sprains, impaired postural control and decreased muscle strength of the hip and ankle joint [14]. Regarding the development of CAI, Doherty et al. [15] identified a couple of risk factors including an inability of drop landing or jumping within two weeks of the initial LAS injury as well as a poorer dynamic postural control and lower levels of self-reported function six months after the initial LAS injury. For treating patients with CAI, Donovan and Hertel [10] developed an evidence-based rehabilitation paradigm that takes into account the major functional limitations typically associated with CAI. These impairments were divided into four different assessment domains including deficits in range of motion, strength, postural control or balance and functional tasks that enable targeted neuromuscular training therapy based on individual deficiencies. Miklovic et al. [16] suggested that the impairment domains could also be helpful for the treatment of acute LAS, since patient suffer from similar limitations that are mostly not adequately addressed in the acute or sub-acute phase. Consequently, the authors recommend considering these functional deficits already at an early stage during rehabilitation after an acute LAS to prevent persistent or even chronic symptoms. However, this concept has not yet been empirically proven within a comprehensive approach. In addition, a large proportion of patients do not receive supervised targeted rehabilitation after acute LAS [16, 17], although there are evidence-based recommendations for the effective treatment and prevention of acute and recurrent sprains, such as early mobilization and exercise therapy, and ankle bracing [18–20].

Therefore, the aims of this four-year retrospective study were firstly to determine the CAI rate depending on age and sex, and secondly to identify possible determinants or contributing factors (i.e., modality of therapy, beginning of therapy) for developing CAI and negative long-term consequences. Both are relevant goals to evaluate and possibly optimize current treatment strategies after incurring acute LAS.

## Methods

### Study design

**Fig. 1:**
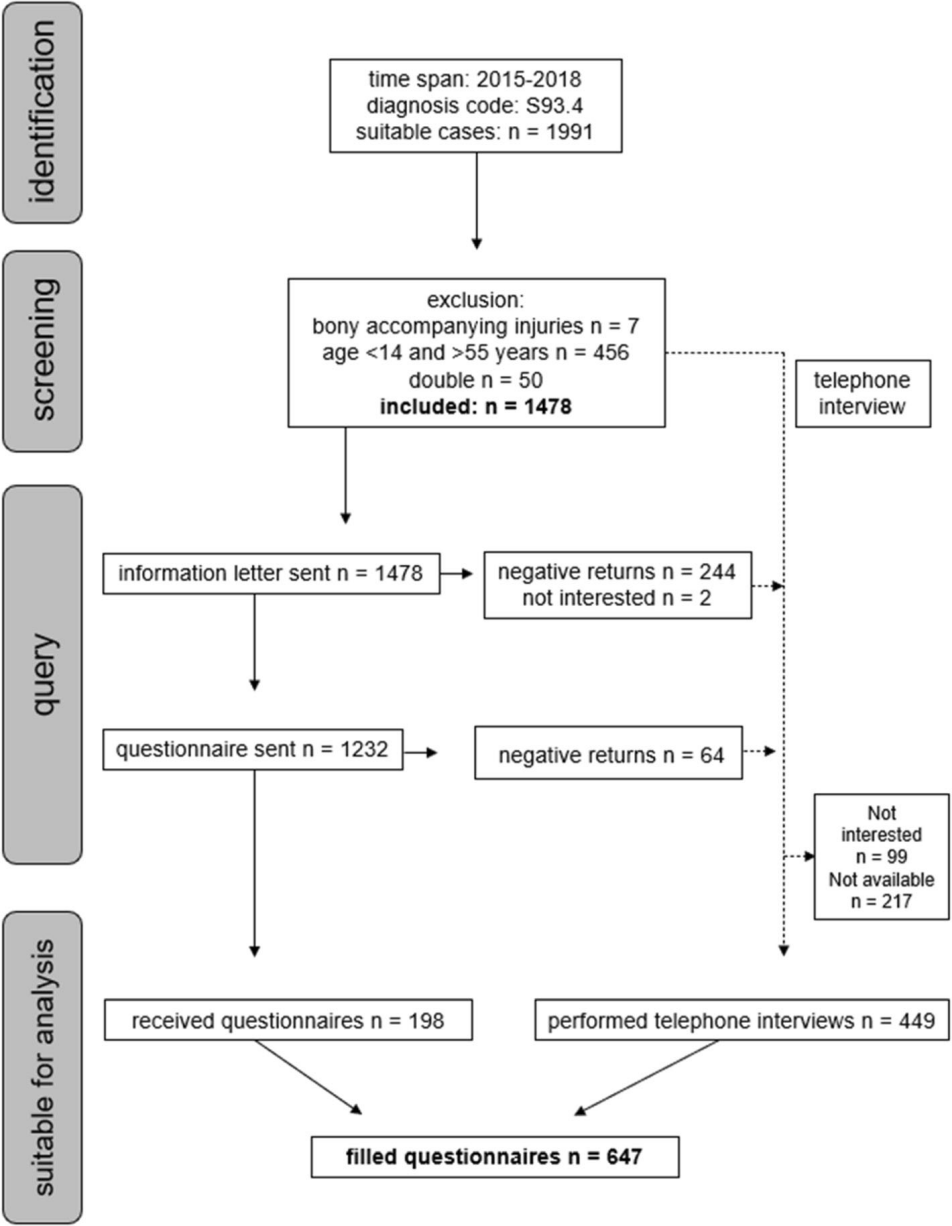
Flowchart of the study design

A retrospective study design was used (Fig. 1). Between 2015 and 2018 we applied the diagnostic code “sprain of ankle” (ICD S93.4) to identify relevant cases from the database of the BG Klinikum Duisburg, Germany. Participants between the ages of 14 to 55 years were included. Valid ankle sprains were defined as acute ankle sprains with no accompanying bone injuries. Cases that did not meet these criteria were excluded. The used criteria were selected due to the recommendations of the International Ankle Consortium [14]. A total of 1478 cases were detected that matched the inclusion criteria.

All patients of the identified cases received a postal questionnaire containing the Tegner-Score and patient-related outcome measures (PROMs) including the Cumberland Ankle Instability Tool (CAIT) and the Foot and Ankle Disability Index (FADI). Additionally, there were questions about the received modality of therapy (i.e., orthosis, physiotherapy and exercise therapy), the beginning of therapy (i.e., immediate start, 1–4 weeks, > 4 weeks or no received therapy), as well as the number of recurrent sprains (i.e., no recurrent sprains, 1-3x, 4-5x). The appropriate answers were marked with a cross by the patients.

The Tegner-Score aims to determine the level of physical activity in patients using different grades on a numerical scale. Its values range from zero to ten, with zero representing being bedridden and ten representing doing competitive sport at a professional level [21]. CAIT and FADI are validated questionnaires to get a subjective insight into regional functional impairments following LAS. The CAIT consists of 9 items measuring the severity of functional ankle instability during the performance of different activities or tasks. The total score ranges from 0 to 30 with 0 representing a painful and strongly instable ankle during low-intense everyday activities and 30 representing a pain-free and subjectively stable ankle even during more intense physical activities. Furthermore, the CAIT is used as a tool to differentiate between individuals suffering from CAI or not by using a pre-defined cut-off score with CAIT ≤ 24 indicating CAI [22]. The FADI assesses functional limitations of the ankle, consisting of 26 items with the possibility to rate the grade of limitation. It is reported as a percentage of the highest possible score [23, 24]. Due to the low number of valid questionnaires received, an additional phone interview was performed. Because of economic reasons, the phone interview included only the PROMs, CAIT and FADI.

### Participants

The received questionnaires (*n* = 198) and additionally performed phone interviews (*n* = 449) led to a total of 647 completed datasets. The sample consisted of 381 male and 266 female participants. Participants were divided into three age groups (14 to 25, 26 to 40 and 41 to 55). Furthermore, they were divided into a CAI and non-CAI group according to the predefined cut-off-score of CAIT ≤ 24, representing at least one out of three selection criteria for having CAI based on international consensus guidelines [22]. Subjects with a CAIT score ≤ 24 points were designated as having CAI, whereas subjects with a CAIT score > 24 points were designated as having non-CAI. Sample characteristics with regard to their level of activity can be seen in Table 1. The study was approved by the local ethics committee (Ärztekammer Nordrhein, 2,018,363) and was conducted in accordance with the Declaration of Helsinki. All participants provided written informed consent.

**Table 1:** Levels of activity separated by age groups

Age group	Tegner-Score	Corresponding level of activity
**All**	6	Recreational sport: tennis, badminton handball
**14–25 years**	7	Competitive sport: Tennis, track and field, handball Recreational sport: soccer
**26–40 years**	5	Heavy physical work Competitive sport: cycling Jogging on uneven terrain
**41–55 years**	4	Medium heavy physical work Recreational sport: cycling Jogging on even terrain

### Statistics

Descriptive statistics using frequency analysis were performed to calculate the percentage CAI rate. Since the FADI was not normally distributed, according to the Kolmogorov-Smirnov-Test (*p* < .05), the Mann Whitney U test was performed to assess differences between the CAI and non-CAI groups in the FADI. Independent t-tests were used to analyze the effects of the reported modality of therapy (yes: received vs. no: not received) on the CAIT score. Relative differences in the beginning of therapy between the CAI and non-CAI group were performed using frequency analysis. A one-way ANOVA was used to determine the effects of the beginning of therapy on the CAIT score and to assess the effects of the number of recurrent sprains on the CAIT score. The Tukey post-hoc test was used for pairwise comparisons between the different starting times of therapy. Homogeneity of variance was verified with the Levene’s test (*p* > .05). In case homogeneity of variance was violated, a Welch ANOVA using Games-Howell post-hoc analysis was applied. Statistical analysis was done using SPSS Statistics (IBM, Armonk, New York, USA, Version 23.0). Data are presented as mean ± SD unless otherwise stated.

## Results

The Tegner score and the frequency of LAS were highest in the younger age group of 14–25 years (46 %) and gradually decreased with advancing age showing the lowest Tegner score and frequency in the older age group of 41–55 years (25 %) (Tables 1 and 2). The overall CAI rate was 17.3 %. Males were consistently less affected than females over all age groups. The highest CAI rate with 22.7 % was found in the 41–55 years age group (Table 2). The total FADI score significantly differed between the CAI and non-CAI group (80.2 ± 16.5 % vs. 97.7 ± 9.2 %; U = 3674.00, Z = -2.237, *p* < .05).

**Table 2:** Frequency of LAS, absolute CAI rate and its relative sex distribution in different age groups

	Frequency of LAS (%)	CAI rate (%)	Male (%) of CAI rate	Female (%) of CAI rate
**all**		17.3	41.4	58.6
**14–25 years**	45.6	11.7	38.7	61.3
**26–40 years**	29.4	12.4	47.6	52.4
**41–55 years**	25.0	22.7	40.0	60.0

**Fig. 2:**
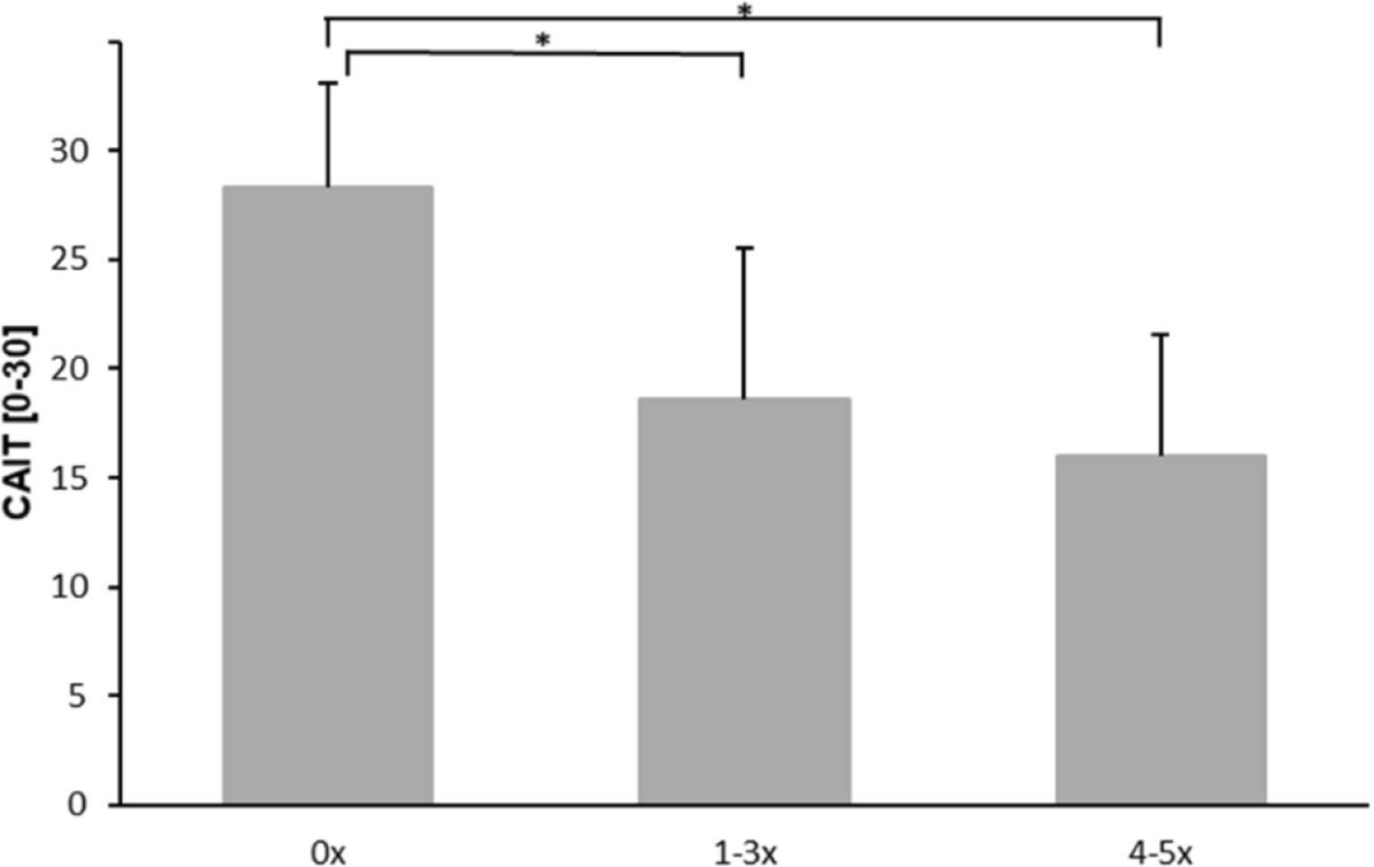
Functional CAIT outcome depending on the frequency of recurrent ankle sprains. *p < .001

The CAIT significantly differed in the reported frequency categories (mean ± SD; no recurrent sprains, 0x: 28.4 ± 4.7, 1-3x: 18.6 ± 6.9 and 3-5x: 16.0 ± 5.5; F(2,562) = 81.379, *p* < .001). Games-Howell post-hoc analysis revealed a significantly higher CAIT score in patients having no recurrent sprain compared to patients having 1–3 recurrent sprains (*p* < .001) or 4–5 recurrent sprains (*p* < .001) (Fig. 2).

**Fig. 3:**
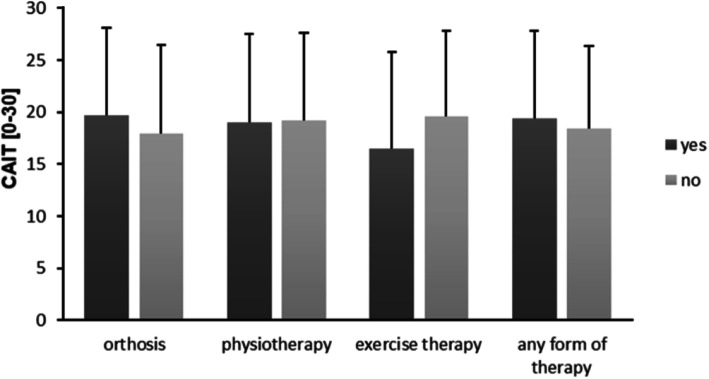
Functional CAIT outcome depending on the modality of therapy

**Fig. 4:**
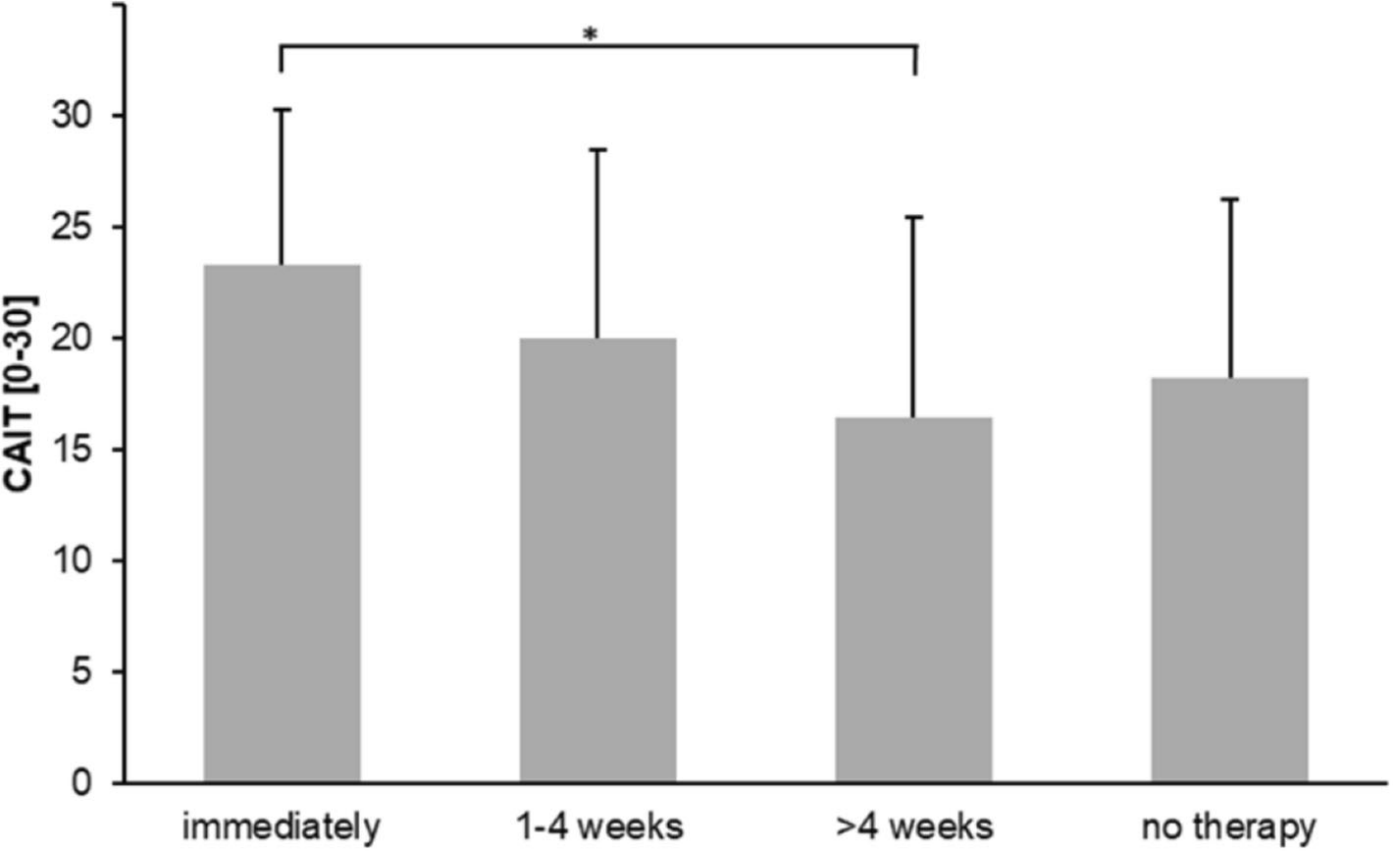
Functional CAIT outcome according to the beginning of therapy. *p < .05

**Fig. 5:**
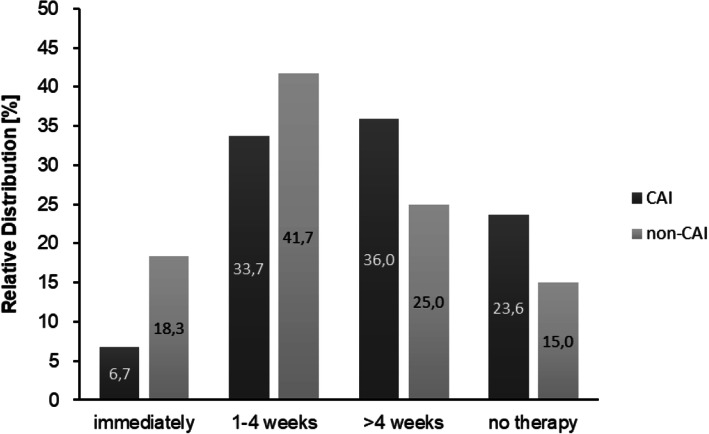
Relative distribution of CAI and non-CAI according to the beginning of therapy

There were no significant differences in the CAIT score between the received and non-received modalities of therapy (Fig. 3). Overall, the mean CAIT descriptively decreased with a later beginning of therapy: immediate start (23.4 ± 6.9), 1–4 weeks (20.0 ± 8.5), > 4 weeks (16.4 ± 9.0), no received therapy (18.2 ± 8.1). ANOVA revealed a significant difference between the starting times of therapy (F(3,145) = 3.34, *p* < .05) showing a higher CAIT score with an immediate start compared to > 4 weeks following acute LAS (*p* < .05) (Fig. 4). At the group level, a higher percentage in non-CAI started their therapy immediately and after 1–4 weeks. By contrast, there was a higher percentage in CAI starting their therapy after more than 4 weeks or receiving no therapy (Fig. 5).

## Discussion

The present study retrospectively analyzed patients with acute LAS treated between 2015 and 2018 in the BG Klinikum Duisburg, Germany. Using PROMs (CAIT & FADI) we collected relevant epidemiological information on the distribution of the CAI rate. We identified a higher CAI rate in females and persons in the age group of 41 to 55 years. We could further show that a later beginning of therapy after acute LAS is associated with increased functional impairments of the ankle. Therefore, early treatment following acute LAS seems to be an effective means in preventing CAI.

The overall CAI rate was about 17 % and thus slightly lower than the CAI rates reported in literature ranging between 20 and 40 % [1, 6, 9, 10, 19]. This could be related to methodological differences in study design such as inclusion criteria and inconsistent terminology according to the definition of CAI. In this study, CAI was classified according to the CAIT score, which is a recommended criteria from the International Ankle Consortium [22]. Our classification is also supported by the FADI since there was a significant difference in the FADI score between the CAI (78.99 %) and non-CAI group (97.05 %).

According to our patients sex we found a higher CAI rate in females (21.2 %) than in males (10.3 %). The females also had higher CAI rates in each of the three age groups. Literature shows that there is in general a higher incidence of ankle sprains in females compared with males (13.6 vs. 6.94 per 1,000 exposures) [4]. This is supported by a higher CAI rate for females in sport (female athletes 32 % vs. male athletes 17 %) [25]. Yet there is no explanation for this prevalence, but there are several assumptions such as increased ankle laxity, increased range of motion, decreased dorsiflexion strength or decreased postural control in females which may contribute to these injuries [26, 27]. Additionally, we speculate that the reduced financial and sportsmedical support in female professional sport compared to their male counterparts might also be a contributing factor. Derived from this, females should focus more on specific prevention programs (e.g. focussing on balance, mobility, strength) to reduce the occurrence of LAS [28].

Doherty et al. [4] state that the incidence of LAS appear to decrease with age. Our data is in line with these findings since we detected a higher LAS frequency in younger compared to older age groups (14–25 years: 46 % vs. 26–40 years: 29 % vs. 41–55 years: 25 %). This may be explained by the corresponding level of activity with higher Tegner scores observed in younger (i.e., increased participation in risk sports with stop-and-go characteristics such as soccer) than older populations. Therefore, the implementation of specific prevention routines is recommended prior to sports activity. In contrast, we found a higher CAI rate in older compared to younger age groups (14–25 years: 12 % vs. 26–40 years: 12 % vs. 41–55 years: 23 %). Currently there is no literature investigating the age distribution of CAI. We assume that there is a higher CAI rate with increasing age because older individuals might have a longer history of multiple ankle sprains in their life, which is a major contributing factor for developing CAI [5]. This may provoke adjustments in lifestyle and a reduction in activity levels that could additionally contribute to a greater development of sarcopenia with decreased muscle mass and connective tissue as well as increased impairments in postural or sensorimotor control, causing an increased ankle instability [29–32]. In addition, our data showed that the frequency of recurrent sprains seems to be associated with the degree of subjective ankle instability, since patients who experienced 1–3 and 4–5 recurrent sprains have significantly worse CAIT outcomes than patients having no recurrent sprains. Furthermore, the CAIT score of the groups with 1–3 and 4–5 recurrent sprains is below the cut-off score of the CAIT (CAIT ≤ 24 indicating CAI), respectively. This implicates that LAS as an injury should be taken seriously and that the focus should be on regaining function as early as possible to prevent recurrent LAS.

With regard to the reported treatment modalities, there were no significant differences in the CAIT score between the received and non-received forms of therapy. Thus, based on the conditions of this study, the functional CAIT outcome does not seem to be generally determined by a specific modality of therapy. As this was a surprising result, we assumed that other factors might play a decisive role on subjective ankle joint function. In this respect, we found that the CAIT score significantly differed between patients receiving their therapy immediately and those who received therapy after more than four weeks. The latter had a worse outcome because of the delayed start of the therapy. This is supported by previous research showing superior treatment effects on subjective ankle function and prevention of CAI in patients with early functional bracing and exercise therapy compared to short-term immobilization [1, 11, 18, 19, 33]. Furthermore, there was a higher percentage of patients in the non-CAI group receiving immediate therapy after acute LAS and, in contrast, a higher percentage of patients in the CAI group receiving therapy after more than four weeks or no therapy at all following acute LAS. We assume that the beginning time of therapy can be seen as an important determinant or contributing factor, respectively, associated with the development of CAI. It seems the later the beginning of therapy after acute LAS the worse the functional outcome.

### Limitations

To increase the number of valid PROMs we performed an additional telephone interview. The individual communication might have influenced the perception and response behavior of patients which could have affected the outcome variables. As this was a retrospective study, there is generally a susceptibility to errors since the data might be biased due to the patient’s inaccurate recollection of events. That is why relevant information about the quality, intensity, duration and frequency of treatment after acute LAS is currently missing, since we decided to ask more simple questions to improve data quality. However, knowledge of the missing data could have affected the study results. The assignment into a CAI and a non-CAI group is based on only one selection criterion, the cut-off score of CAIT ≤ 24 for the definition of CAI. Future studies investigating CAI patients should therefore consider all three criteria recommended by the International Ankle Consortium [22]. Given diagnoses cannot be proved retrospectively on the basis of the etiology and completeness as well as the grading of the severity of the injury. However, this should be taken into account in future and especially prospective studies.

## Conclusions

According to this study, females and older age groups (41–55 years) have a higher risk for developing CAI which implies to focus on specific prevention or therapy programs improving ankle function. Moreover, patients reporting a later start of therapy after acute LAS (> 4 weeks) have a worse functional CAIT outcome, irrespective of the received treatment modality. Thus, a delayed beginning of therapy following acute LAS seems to be an important determinant associated with the development of CAI. A further contributing factor for CAI, suggested by literature, is the number of recurrent sprains which could also be supported by our data. Therefore, we highly recommend an early start of functional therapy after acute LAS in the future to minimize the LAS recurrence rate and the development of CAI. The therapy should be guided by four impairment domains, identified in patients with CAI [16]. These domains consist of range of motion, strength, postural control, and functional tasks. Further research in this area is needed to empirically evaluate the effectiveness of this treatment concept aiming to reduce the CAI rate.

## Data Availability

The datasets generated during and analyzed during the current study are not publicly available due to maintaining control of further data usage or data inclusion in future research projects. In general, we are keen to share our datasets, which is why they are available from the corresponding author on reasonable request.

## Abbreviations

CAI: Chronic ankle instability
CAIT: Cumberland ankle instability tool
FADI: Foot and ankle disability index
LAS: Lateral ankle sprain
PROMs: Patient related outcome measures
